# LncLocation: Efficient Subcellular Location Prediction of Long Non-Coding RNA-Based Multi-Source Heterogeneous Feature Fusion

**DOI:** 10.3390/ijms21197271

**Published:** 2020-10-01

**Authors:** Shiyao Feng, Yanchun Liang, Wei Du, Wei Lv, Ying Li

**Affiliations:** 1 Key Laboratory of Symbol Computation and Knowledge Engineering of Ministry of Education, College of Computer Science and Technology, Jilin University, Changchun 130012, China; fengsy18@mails.jlu.edu.cn (S.F.); ycliang@jlu.edu.cn (Y.L.); weidu@jlu.edu.cn (W.D.); 2 Zhuhai Laboratory of Key Laboratory of Symbol Computation and Knowledge Engineering of Ministry of Education, Zhuhai College of Jilin University, Zhuhai 519041, China; luwei@jluzh.edu.cn

**Keywords:** subcellullar location, multi-source features, the binomial distribution-based filtering, logarithm-distance of Hexamer

## Abstract

Recent studies uncover that subcellular location of long non-coding RNAs (lncRNAs) can provide significant information on its function. Due to the lack of experimental data, the number of lncRNAs is very limited, experimentally verified subcellular localization, and the numbers of lncRNAs located in different organelle are wildly imbalanced. The prediction of subcellular location of lncRNAs is actually a multi-classification small sample imbalance problem. The imbalance of data results in the poor recognition effect of machine learning models on small data subsets, which is a puzzling and challenging problem in the existing research. In this study, we integrate multi-source features to construct a sequence-based computational tool, lncLocation, to predict the subcellular location of lncRNAs. Autoencoder is used to enhance part of the features, and the binomial distribution-based filtering method and recursive feature elimination (RFE) are used to filter some of the features. It improves the representation ability of data and reduces the problem of unbalanced multi-classification data. By comprehensive experiments on different feature combinations and machine learning models, we select the optimal features and classifier model scheme to construct a subcellular location prediction tool, lncLocation. LncLocation can obtain an 87.78% accuracy using 5-fold cross validation on the benchmark data, which is higher than the state-of-the-art tools, and the classification performance, especially for small class sets, is improved significantly.

## 1. Introduction

Only 2% of the transcriptional products are translated into proteins, and the remaining 98% are non-coding RNAs. In a long period, researchers ignore the role of non-coding RNAs in life activities, which are even considered as junk in the evolution. However, with the rapid development of life science, more and more noncoding RNAs are proved to play vital roles in human gene transcription regulation, cell growth, differentiation, breeding, and other life activities [1,2,3,4]. The research on non-coding RNAs mainly focuses on micro RNAs (miRNA), circular RNAs (circRNA), small interfering RNAs (siRNA), PIWI-interacting RNAs (piRNA), and long non-coding RNAs (lncRNA). The lncRNAs, with a length of more than 200 nt noncoding RNA, which are the majority of noncoding RNAs, often play essential roles in life activities and highly relate to various disease, including neurological disease and tumors [5,6,7,8,9]. At present, the research on lncRNAs mainly starts from the two aspects of functional acquisition and functional deficiency [10,11,12,13]. Overexpression and RNA agonists can be used for functional acquisition verification [14,15], while RNA inhibitors, antagonists, and promoter knockout are suitable for functional deletion. However, not all experimental methods are applicable to lncRNAs. For example, to be interfered with by RNA inhibitors, lncRNA should be located in the cytoplasm. However, lncRNAs are selectively distributed in the nucleus and cytoplasm. Cells are divided into different organelles; various organelles have different divisions of labor and are responsible for the activities of cells with different functions, thus the information of subcellular localization of lncRNAs can contribute to its function. Therefore, prediction of the subcellular localization of lncRNAs is very significant. Determining lncRNAs in the distribution of various organelles can effectively contribute to understanding its functions and molecular mechanisms of lncRNAs. However, existing experiment methods are both time-consuming, expensive, and laborious, so it is necessary to study the prediction of lncRNA subcellular localization.

In order to better study subcellular localization of lncRNAs, many databases have been provided. Zhang et al. constructed a RNA subcellular location database, RNALocate, in which there are 1361 lncRNAs among 37,700 ncRNAs of multiple species [16]. The LncATLAS database [17] is the first database to specifically include lncRNA subcellular localization data, which is based on high-throughput sequencing data of 15 cell lines and includes 6768 lncRNA data from GENCODE annotation. By studying the localization of lncRNAs in gastric cancer cells, Cheng and Leung et al. (2018) confirmed the relationship between the localization of lncRNA cells and gastric cancer [18]. Subsequently, Feng et al. first proposed a computational method to predict the subcellular localization of non-coding RNAs on kinetoplast, mitochondria, and chloroplast [19]. Currently, there are limited computational prediction methods for the subcellular localization of lncRNA, mainly including multi-classification of lncLocator and iLoc-lncRNA, which contain five subcellular localization regions and four subcellular localization regions, respectively, and DeepLncRNA based on binary classification, which contains two subcellular localization regions. Zhen et al. extracted the K-mer features from the sequence, and then use the stacked automatic encoder to learn higher level features from the K-mer features. After using the oversampled data balance method, these features were fed to the integrated classifier composed of random forest (RF) and support vector machine (SVM), and finally the prediction tool lncLocator was derived [20]. Su and Huang et al. constructed a predictor named iloc-lncRNA [21] to predict the subcellular localization of lncRNA. Through the binomial distribution screening method in pseudo k-tuple nucleotide composition, the filtered K-mer data were fed to SVM [22].

In this study, we propose a novel multi-source heterogeneous feature fusion computational tool to predict the subcellular location of lncRNAs, lncLocation.

First, to capture the panorama of lncRNA subcellular localization information from multiple perspectives, we construct multi-source features of lncRNAs, including sequence composition features, basic lncRNA features (ORF length and coverage, the EDP of ORF, mean hexamer score, GC content of the non-ORF region, and Fickett nucleotide features), physical-chemical properties, and multi-scale secondary structural features. Second, to further improve the representation and reduce the impact of data imbalance, a computational framework of multi-source feature fusion is proposed to integrate deep feature learning based on an autoencoder, and hybrid feature selection based on recursive feature elimination and binomial distribution filtering. The 8-mer feature is further processed with the filter filtering method based on a binomial distribution, and the other features are further processed with the recursive feature elimination algorithm after further learning using an autoencoder. Third, by comprehensive experiments on various machine learning models and features, the optimal model lncLocation is determined. Then, lncLocation is compared to existing state-of-the-art methods for lncRNA subcellular localization prediction, which shows higher prediction performance, especially for the subcellular location with a small size. Furthermore, in the application case of lncLocation, we make a whole human-genome prediction of lncRNA subcellular localization using lncLocation, and further analyze the distribution of human lncRNA in four organelles. Finally, for convenience, an online web server is developed for researchers to use.

## 2. Results

### 2.1. The Effectiveness of Different Features

The model integrates features from multiple sources.

Different machine learning models were used to evaluate the performance of different features. For a comprehensive model comparison, we constructed different machine learning models from traditional machine learning models, including logistic regression, random forest, and support vector machines, then integrated learning methods, such as XGBoost and LightGBM, to deep learning models, including deep neural networks (DNNs) and convolutional neural networks (CNNs).

Here, we adopted a layered feature combination scheme. Compared with a single feature, the layered feature combination is more effective and efficient.

(1) The different types of features extracted by the above methods were fed to different traditional machine learning models, respectively. The performance of the different features under different machine learning models is listed in Table 1.

From Table 1, it can be seen that the physicochemical properties of sequences and multi-scale structural features have a strong ability to classify lncRNA subcellular localization, which achieve an accuracy of 70.18% and 70.42%, respectively, on LightGBM. Moreover, the multi-scale structure features can obtain the highest accuracy rate of 71.69% on SVM. In general, all the features can obtain good results on SVM. At the same time, it can be concluded that each feature plays a certain role in the localization and recognition of lncRNA subcellular location.

(2) In order to further integrate features, reduce the redundancy of features, and improve the classification performance, we grouped the features into fea.Tuple containing raw 8-mer features, and fea.Bio consisting of the remaining features, respectively.

For fea.Bio, the new fea.Bio was obtained after feature screening. In order to further compare the performance of different feature groups. XGBoost was used on the benchmark dataset. XGBoost can automatically learn the optimal missing value according to the training loss and more effectively process different types of sparse patterns in the data, which is more stable for different batches of training [23,24,25].

While performing feature filtering on the fea.Bio, we tested the intermediate results of the screening. The test results show that, compared with the sequence features extracted from the original sequence, the advanced features reprocessed by the autoencoder have a higher representativeness of the classification target. The former obtained a 70.99% accuracy on the test model, while the advanced features coded in 32 and 64 dimensions obtained a 74.53% and 74.02% accuracy, respectively. Although the representation of a single set of features on the same test model is different, each characteristic has different attributes on the classification of the target, so sequence features and secondary features were fused together and further filtered using recursive feature elimination algorithms based on XGBoost. By testing the screening features, the accuracy rate was 78.83%. It can be observed that the final screening feature significantly improves the test model results.

**Figure 1:**
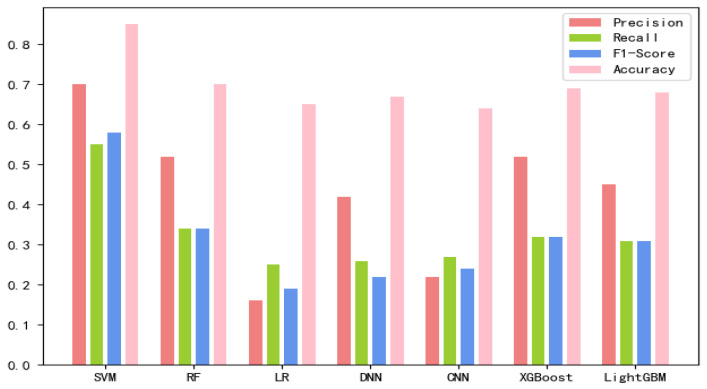
New fea.Tuple training results on each model.

**Figure 2:**
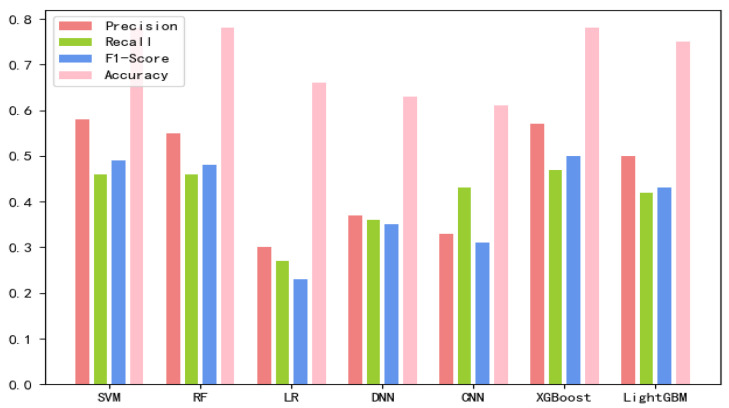
New fea.Bio training results on each model.

**Figure 3:**
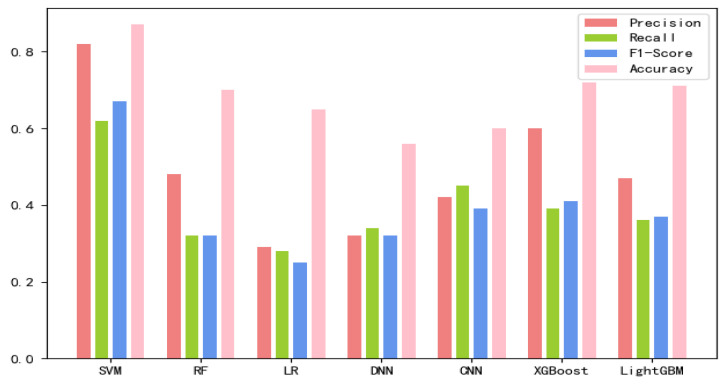
Connecting new fea.Tuple and new fea.Bio training results on each model.

The 8-mer features by using the screening method were obtained, named new fea.Tuple [21]. We applied new fea.Bio and new fea.Tuple to the training of each model, then combined them into a group of features for training, and finally obtained three control groups of each training model. The final training data are shown in Figure 1, Figure 2 and Figure 3 The values of the training results can be obtained from the Supplementary Materials Tables S1–S3.

These models underwent careful parameter adjustment. Traditional machine learning models and integrated learning use the grid search strategy [26], while, due to the large number of parameters of deep learning models, the random search method is used.

Based on the comparison of various test results, the SVM classification combining both new fea.Tuple and new fea.Bio has the best performance, so lncLocation was constructed. Here, the radial basis kernel function is used as the kernel function, and the one-versus-one strategy is used as the multi-classification strategy. In this case, the new fea.Tuple and new fea.Bio are used in the feature group. The grid search strategy is used to optimize the regularization parameter C and the kernel width parameter γ, where the search space was set as [2^−5^, 2^10^] and [2^0^, 2^−15^], respectively. The values of parameter C and parameter γ are finally obtained. In the case of 5-fold cross-validation, the total accuracy of the model was 87.78%, and the total accuracy was 89.69% on an independent set of validation that accounted for 20% of the total sample. We adopted the strategy of stratified sampling to ensure that the proportion of each subset in the cross-validation and each subset in the separate validation set was consistent with the benchmark set, so as to make the final verification result more objective and accurate.

### 2.2. Comparison to Existing Methods

Finally, lncLocation was compared with some existing methods on the benchmark data with 10-fold cross-validation. For comparison, we also list the test results of lncLocator and iLoc-lncRNA. Because DeepLncRNA is a two-category classifier, its comparison results are omitted here. The comparison results are shown in Table 2. As can be seen from Table 2, lncLocation is more advantageous in identifying subsets which possess a small number, and gets better results in all subsets except for the nucleus. Compared with iLoc-lncRNA, lncLocation increased the precision of cytoplasm by 25.59%, the recall of cytoplasm by 0.94%, the precision of ribosome by 0.17%, the recall of ribosome by 19.45%, and the recall of exosome by 49.98%.

### 2.3. Web Server and User Guide

**Figure 4:**
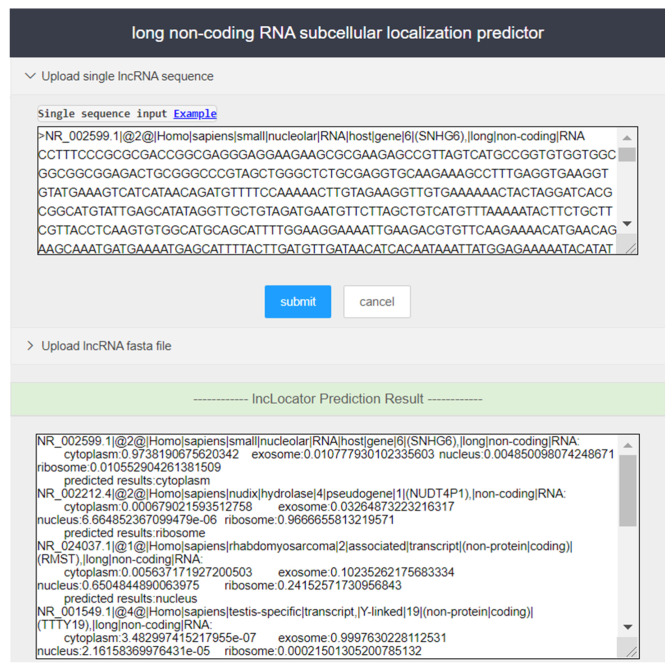
Input and output part of the screenshot of the lncLocation web server.

A user-friendly tool can greatly improve the efficiency of researchers. While providing open-source prediction tools, we constructed a web server with a friendly interface. The server core is based on python 3.7 and uses open source third-party libraries, including scikit-learn, pandas, numpy, and tensorflow. A detailed list of third-party tools and corresponding version numbers can be found in the lncLocation package’s instructions (https://github.com/FengSY-JLU/Core-lncLocation/) and the server usage guide is given as follows:

Step 1. Access the lncLocation website (http://lnclocation.nat100.top/) in the browser, and users can see the server page as shown in Figure 4.

Step 2. Enter lncRNA sequence to be predicted in the input box of the page (in fasta format), or upload the user sequence file in fasta format, and click ‘Submit’ to run lncLocation for prediction of subcellular localization. Users can enter the sample sequences for testing by clicking the ‘Example’ button.

Step 3. In the output box of the page, the prediction results of lncRNAs are provided.

### 2.4. A Prediction on the Human Genome

**Figure 5:**
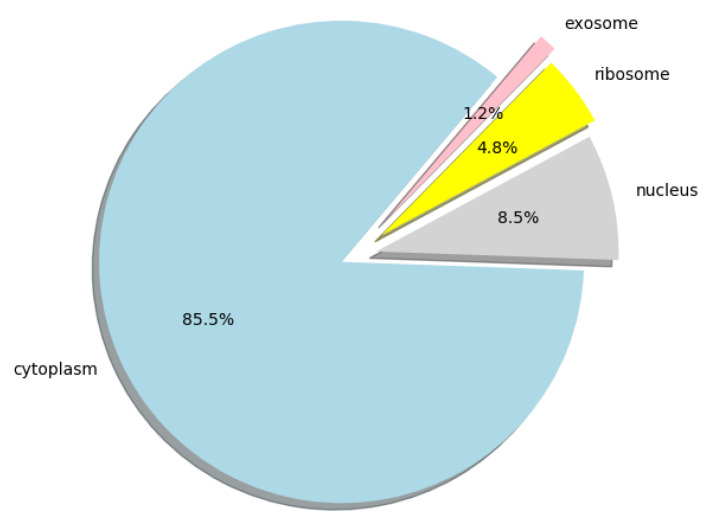
Pie chart of the distribution ratio of lncRNA in four organelles.

Furthermore, we used lncLocation to predict the lncRNA subcellular location at the human-genome scale, and obtained the distribution of 25,405 human lncRNA sequences in the four organelles. All the predicted results can be obtained from Supplementary Materials File S2, the human lncRNA dataset we used can be obtained from Supplementary Materials File S3, and the distribution ratio of lncRNA in the four organelles is shown in Figure 5.

## 3. Discussion and Conclusions

Due to the limited data of lncRNA subcellular localization verified by experiments, and the data of different subcellular localization types being unbalanced, there are few labeled lncRNA subcellular data, resulting in the imbalance between different subcellular location data. The problem of multi-classification of small samples has always been a difficult problem in the field of machine learning. In order to solve this problem, we proposed a multi-source heterogeneous feature extraction scheme for the prediction of lncRNA subcellular location, lncLocation. The experimental results demonstrate the effectiveness of the system and that the accuracy of the model is higher than that of the existing multi-classification lncRNA subcellular localization tools. Moreover, the classification performance of small-scale data subsets was also improved significantly, indicating the effectiveness of multi-source heterogeneous feature fusion in lncRNA subcellular localization. Different feature screening methods are used respectively for feature subsets with large differences to improve the representation capability of smaller data subsets. A single feature extraction scheme cannot fully obtain the subcellular localization content of lncRNA sequence. Feature extraction requires multiple kinds of hierarchical structures. Multi-source features can further describe the sequence information from different aspects, and hierarchical features can further reveal the intrinsic nature of the information. Through the extraction of multi-source heterogeneous features and the secondary processing-based autoencoder, the sequence representation ability was significantly improved, which shows that the autoencoder can indeed learn higher level representations and further enhance the capacity of the presentation of features that cannot be extracted manually. In our experiments, the recursive feature elimination for further screening was demonstrated to efficiently reduce the redundancy, and further improve the generalization ability of the model, as the prediction precision of cross-validation increased from 74.53% to 78.83%.

The different communication of different types of features and classifiers show different prediction performances. Even for the same type of feature, the performances of different classifiers vary greatly. Due to the small size of the sample, deep learning models are not better than traditional machine learning models. This may be consistent with Occam’s razor principle. Among all possible models, the best model is one that can explain the known data well and is very simple, which is the model that should be selected [27,28]. In this study, the filtered k-tuple features were combined with the multi-scale structure features, which was selected to construct lncLocation, and SVM showed the best performance, compared to other combinations. To some extent, this demonstrates that the multi-scale feature and k-tuple feature respectively contain different contents of lncRNA sequences on the target of subcellular localization, which can effectively improve the representation ability of each other.

The feature extraction and integration scheme employed in this study can be directly used for other RNA sequence analysis. In the future, more types of features should be extracted and integrated by the multimodal machine learning models for prediction of lncRNA subcellular location, such as lncRNA–protein associations and lncRNA expression data, to improve the prediction performance and provide an effective and practical tool.

## 4. Materials and Methods

**Figure 6:**
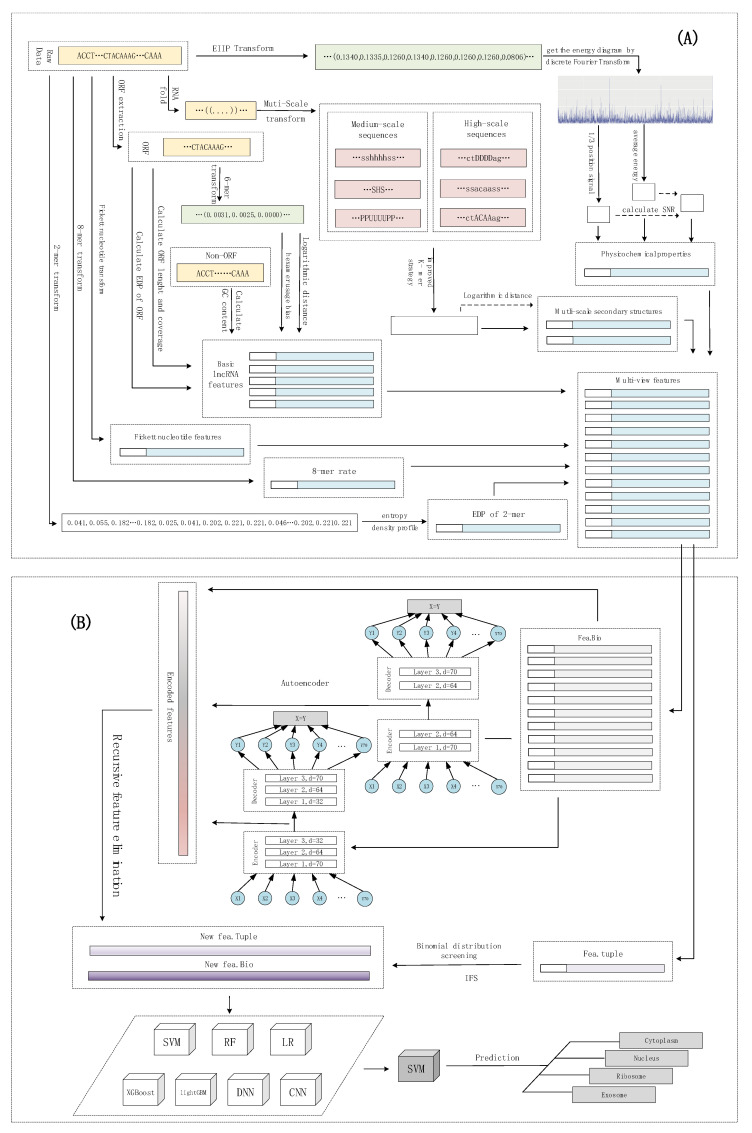
The flowchart of lncLocation. (**A**) Multi-source feature extraction; (**B**) Feature learning and model selection.

The subcellular localization of lncRNAs can be considered as a multi-classification problem. lncLocation was constructed by the following five steps. The first step was to extract multi-source features, including features based on the k-mer composition frequency (raw 8-mer, the EDP of 2-mer), basic lncRNA features, physicochemical properties, and multi-scale secondary structure features. After using the autoencoder to extract advanced features, a process, including the recursive feature elimination (RFE) method and regularization method, was introduced to select the optimal features and eliminate effects of dataset imbalance. For the sequence composition features, the binomial distribution method and iterative feature selection (IFS) method were used to extract the most informative 8-mer composition features. By comparing different multi-source feature combinations and different classifiers, including traditional machine learning models and deep learning models, the most suitable feature combinations and classifier were selected to construct lncLocation. Finally, lncLocation was validated as being efficient and effective for the prediction of lncRNA subcellular location on the benchmark dataset, compared to other existing state-of-the-art methods. The flowchart of lncLocation is shown in Figure 6.

### 4.1. Benchmark Dataset

The dataset was downloaded from RNALocate (http://www.rna-society.org/rnalocate/), which is a comprehensive database focusing on collecting RNA localization information. Through manual screening, 986 lncRNA sequences with annotated subcellular localization information were obtained. In order to eliminate the impact of redundant sequences, the CD-HIT [29] program with a cutoff of 80% was used to get rid of the redundant sequences. In total, 653 lncRNA non-redundant sequences were finally obtained, including 4 subsets, 424 samples of cytoplasm, 156 samples of nucleus, 43 samples of ribosome, and 30 samples of exosome. The data used can be obtained from Supplementary Materials File S1. The detailed statistics of the dataset used in this paper are listed in Table 3.

### 4.2. Multi-Source Feature Extraction

Multi-view features, including features based on the k-mer composition frequency, basic lncRNA features, physicochemical properties, and secondary structure features, were extracted.

#### 4.2.1. K-Tuple Features

The distribution of adjacent bases is different in different non-coding RNA transcripts [30], and k-tuple is the most common method to obtain this distribution difference. K-tuple is a virtual sequence fragment, which is widely used to encode RNA by cutting the sequence into a specific length of nucleotide subsequence and analyzing its contents [31]. A specific lncRNA sequence S can be described as:

(1) $$S=\left(N_{1}N_{2}N_{3}\cdots N_{m}\right),$$

where N represents the four different bases (i.e., A, C, G, T) in the lncRNA sequence, and m refers to the length of sequence S. The pseudo amino acid composition (PseAAC) method [32], and the pseudo k-tuple nucleotide composition (PseKNC) [22] method are proposed to transform the base sequence into a real-value vector.

A k-mer pattern contains $4^{k}$ entries. By counting the number or frequency of each k-mer entry, it is finally converted into a vector of $4^{k}$ dimensions. This frequency continuous conversion method preserves more fully the internal information of the sequence, and the k-mer frequency has important biological significance [33]. Some studies have revealed the unique evolutionary mechanism of 8-mer:

(2) $$V\left(S\right)={\left[K_{1}K_{2}K_{3}\cdots K_{65536}\right]}^{T}.$$

As raw 8-mer has a large characteristic dimension and a large number of redundant features, in order to improve the performance of the model and remove the redundancy, further feature learning and selection are usually carried out, such as using a stacked autoencoder to extract high-level abstraction of lncRNA sequences from raw k-tuple features [20], and using the binomial distribution to screen raw k-tuple features [21].

The entropy density profile (EDP) model uses Shannon artificial language to describe a fixed-length sequence, which is a global statistical description of a given sequence. Like the k-Tuple model, EDP also extracts features from the global perspective of the sequence and constructs corresponding dimensional real-value vectors to describe sequence information. The use of EDP is based on both the amino acid composition and 2-mer patterns [34,35]. The number of k-tuples depends on k, and there are 16 2-mer patterns (the k power of the number of nitrogen base), and finally, the sequence is converted to a 16-dimensional vector. There is a reasonable deduction that the EDP phase space contains bias between the cluster of lncRNA sequences on different subcellular organelles [36]. Thus, the entropy density profile of 2-mer can be described as:

(3) $$S_{i}=-\frac{1}{H}f_{i}\;log\;f_{i},$$

where $H=-\overset{16}{\underset{i=1}{\sum }}f_{i}\;log\;f_{i}$ is the Shannon entropy, and $f_{i}$ is the abundance of the *i*th 2-mer [37].

#### 4.2.2. Basic lncRNA Features

With the development of deep ribosomal sequencing (RIbo-Seq), PhyloCS, and mass spectrometry, more and more evidence shows that although the ORF of lncRNA does not have protein coding ability, some ORF of lncRNA can encode small peptides [38], which indicates that the contents contained in the open reading frame are related to different functions among different lncRNAs. ORF length is the longest length of entry range from the start codon (ATG) to the end codon (TAG, TAA, or TGA), and the coverage refers to the ratio of ORF to full entry, which can be described as follows:

(4) $$COV_{orf}\left(s\right)=\frac{ORF\left(S\right)}{l\left(S\right)},$$

where ORF(S) and l(s) refer to the ORF length and full length of the sequence S, respectively. EDP of ORF is used to quantify the ability of a sequence of ORF to encode small peptides:

(5) $$EDP_{orf}\left(s\right)=-\frac{1}{H}c_{i}\;log\;c_{i},$$

where $H=-\overset{20}{\underset{i=1}{\sum }}c_{i}\;log\;c_{i}$ is the Shannon entropy, and $c_{i}$ is the *i*-th codon that can be translated into amino acid.

The hexamer usage bias of the ORF length is the more discriminating feature [39]. We used logarithmic likelihood ratios to measure the difference in the use of hexamers between sequences belonging to a particular subset and other subsets, which could more effectively capture the difference between sequences of different subsets [40]. The hexamer usage bias μ(s) is described as the following formula:

(6) $$\mu \left(s\right)=\frac{1}{m}\sum_{i=1}^{m}log\;\frac{F_{j}\left(H_{i}\right)}{F\left(H_{i}\right)},$$

where $F_{j}\left(H_{i}\right)$ refers to the in-frame frequency of the *i*-th hexamer in the *j*-th subset, and $F\left(H_{i}\right)$ refers to the in-frame frequency of the *i*-th hexamer among all the rest of the subsets.

At the same time, we considered the frequency of Hexamer and introduced a method to quantify the distance of Hexamer on different subsets: Logarithm-distance of Hexamer in LncFinder [41]. Considering the deviation of the data of the quad classification problem here, the two largest subsets (cytoplasm and nucleus) were considered and the logarithmic distances of the four subsets were calculated, respectively, which can be described as follows:

(7) $$log\;Dist_{Cyto}=\frac{1}{n}\sum ln\frac{freq_{seq}\left(i\right)}{freq_{cyto}\left(i\right)},\;\;\;\left(i=1,2,\ldots .,4^{k}\right),$$

(8) $$log\;Dist_{nuc}=\frac{1}{n}\sum ln\frac{freq_{seq}\left(i\right)}{freq_{nuc}\left(i\right)},\;\;\;\;\left(i=1,2,\ldots .,4^{k}\right),$$

(9) $$Ratio_{Dist}=\frac{log\;Dist_{Cyto}}{log\;Dist_{nuc}},$$

where $freq_{seq}\left(i\right)$, $freq_{cyto}\left(i\right)$, and $freq_{nuc}\left(i\right)$ are the i-th hexamer frequency of the unevaluated sequence, cytoplasm, and nucleus, respectively; and n refers to the total number of the hexamer in the sequence. The ratio of the distance $Ratio_{Dist}$ can be calculated from $log\;Dist_{Cyto}$ and $log\;Dist_{nuc}$.

Meanwhile, the non-ORF part of the sequences was also considered on account of 5′UTRs and 3′UTRs of a transcript showing a significant capacity in lncRNA identification. Due to the difference in the GC content between 5′UTRs and 3′UTR [42], the coverage of 5′UTRs and 3′UTRs, as well as the GC content in 5′UTRs and 3′UTRs, to describe the characteristics of non-ORF regions were all considered. The coverage of 5′UTR is defined in the following:

(10) $$COV_{5’UTR}\left(s\right)=\frac{5’UTR\left(S\right)}{l\left(S\right)},$$

where 5’UTR(S) and l(s) refer to the 5′UTR length and full length of the sequence S, respectively. Similarly, the coverage of 3′UTR can be obtained.

Fickett nucleotide features are simple semantic features, which compute the nucleotide composition and positional frequency of the sequence [43]. Fickett nucleotide features have significant classification efficiency due to differences in the nucleotide content and position in sequence clusters [39]. The position frequency of nucleotides counts the degree to which each base is superior to the others at one position in the subsequence fragment. Nucleotide composition is the percentage of a certain base (i.e., A, C, G, T) of the sequence, and the nucleotide position frequency requires calculation based on the value of the base at each position in the sequence fragment. For example, the position value of A can be expressed as:

$$A_{1}=Number\;of\;A^{\prime }s\;in\;positions\;1,\;4,\;7\ldots ,$$

$$A_{2}=Number\;of\;A^{\prime }s\;in\;positions\;2,\;5,\;8\ldots ,$$

$$A_{3}=Number\;of\;A^{\prime }s\;in\;positions\;3,\;6,\;9\ldots ,$$

(11) $$A_{pos}=\frac{MAX\left(A_{1},A_{2},A_{3}\right)}{MIN\left(A_{1},A_{2},A_{3}\right)+1},$$

where A represents the nucleotide; and $C_{pos}$, $G_{pos}$, and $U_{pos}$ are calculated similarly. The percentage of each base in the sequence also needs to be determined, and eventually each sequence is converted to an 8-dimensional vector. In the original version of Fickett [43], the probabilities of these eight values are further calculated using the lookup table, and the TESTCODE score is calculated using the corresponding weights.

#### 4.2.3. Physicochemical Properties

The electron-ion interaction pseudopotential (EIIP) is used to calculate the energy of delocalized electrons in nucleotides as a new nucleotide coding scheme. Here, we introduced EIIP values as the physicochemical properties of lncRNA sequences. The nucleotide EIIP values obtained from [44] are [A—0.1260; C—0.1340; G—0.0806; U—0.1335].

The lncRNA sequence was converted to the EIIP numerical vector by using the nucleotide EIIP value, which can be denoted as X[N]. The corresponding power spectrum can be obtained by using the discrete Fourier transform:

(12) $$H\left[k\right]=\sum_{n=0}^{N-1}X\left[n\right]e^{\left(-j2\pi kn∕N\right)}{}_{,}\;k=0,\;1,\;2,\ldots N-1,$$

and the corresponding power spectrum is defined as:

(13) $$s\left[k\right]={\left|H\left[k\right]\right|}^{2}.$$

For lncRNAs belonging to different clusters, their spectral energy is also different. Therefore, we used the 1/3 position signal, average energy, and signal-to-noise ratio as the characteristics. The $\bar{E}$ and SNR are described as follows:

(14) $$\bar{E}=\frac{\sum_{k=0}^{N-1}s\left[k\right]}{N},$$

(15) $$SNR=\frac{s\left[\frac{N}{3}\right]}{\bar{E}}.$$

Considering the difference of the EIIP power spectrum between lncRNA sequences, we further conducted a descending order of the power spectrum to calculate the quantile statistics of the power values (Q1, Q2, Q3, minimum, and maximum) in different ranges. The Q1, Q2, maximum, and minimum values of quantile statistics were taken as physicochemical properties for model training.

#### 4.2.4. Multi-Scale Secondary Structures

The secondary structure of lncRNAs is more conservative than the sequence, which is of great significance for lncRNAs function inference of lncRNAs. The secondary structure of RNA plays an important role in a variety of biological functions and is more stable than the features of the primary sequence [45,46]. Here, we used the multi-scale secondary structure feature to further extract the features of the sequence. Multi-scale secondary structure features can portray the structural information from the three levels of stability, sub-elements (SSEs) combined with the pairing condition and structure-nucleotide sequences [41].

The minimum free energy (MFE) shows the structural stability of an RNA, and the secondary structure of lncRNA was obtained through the ViennaRNA package [47] based on the minimum free energy algorithm. LncRNAs in different clusters have different stabilities, which could contain different MFE. Let S[n] and SS[n] represent lncRNA sequences with a length n. SS[n] is marked with a dot bracket notation, that is, here, the bases in the sequence were replaced with a dot bracket notation, the paired bases were replaced with open and close brackets, and the non-paired bases were replaced with dot notation. The sub-elements (SSEs) of lncRNA contains four components, i.e., stem(s), bulge(b), loop(l), and hairpin(h). The SSE full sequence (SSE.Full Seq), one of the secondary structure-derived sequences, can be obtained by replacing nucleotides in the sequence with corresponding SSEs. Successive identical SSEs were marked as one SSE to obtain another derived sequence of the secondary structure, which was named the SSE abbreviated sequence (SSE.Abbr Seq). P (pair) and U (un-pair) were used to replace the bracket and dot in SS[n] to get Paired-Unpaired Seq:

$$Paired-Unpaired\;S\left[n\right]=\{\begin{array}{c} U,\;\;SS\left[n\right]=.\\ P,\;\;SS\left[n\right]\neq . \end{array}.$$

The nucleotide composition of the sequence S[n] was then used to derive three derived sequences of structural characteristics from the secondary structural sequence SS[n] at a high scale level, which were named acgu-Dot Sequence (acguD S[n]), acgu-Stem Sequence (acguS S[n]), and acgu−ACGU S[n]:

$$acguD\;S\left[n\right]=\{\begin{array}{c} D,\;\;\;\;\;\;\;SS\left[n\right]=.\\ S\left[n\right],\;\;SS\left[n\right]\neq . \end{array},$$

$$acguS\;S\left[n\right]=\{\begin{array}{c} D,\;\;\;\;\;\;\;SS\left[n\right]\neq .\\ S\left[n\right],\;\;SS\left[n\right]=. \end{array}$$

$$acgu-ACGU\;S\left[n\right]=\{\begin{array}{c} \begin{array}{c} A,\;\;\;S\left[n\right]=a\;\;\wedge \;\;S\left[n\right]\neq .\\ C,\;\;\;S\left[n\right]=c\;\;\wedge \;\;S\left[n\right]\neq .\\ G,\;\;\;S\left[n\right]=g\;\;\wedge \;\;S\left[n\right]\neq .\\ U,\;\;\;S\left[n\right]=u\;\;\wedge \;\;S\left[n\right]\neq .\\ S\left[n\right],\;\;\;\;\;\;\;\;\;\;\;\;\;\;\;\;\;\;\;\;\;SS\left[n\right]=. \end{array} \end{array}.$$

The sequence of  acguD replaces unpaired nucleotides with *D*, describing the coverage of SSE STEM in the sequence. Similarly, the sequence of acguS describes the coverage of the sequence except for the stem. The sequence of acgu−ACGU stores the nucleotide information of the sequence as well as the SSEs information by distinguishing the nucleotide pairs from unpaired nucleotides in the sequence of upper and lower letters. These three kinds of sequences describe the secondary structure of sequences at a higher level. Then, the improved k-mer strategy [48] and the logarithmic distance of k-mer [41] were used to extract the features of these structure-derived sequences, and each sequence was finally transformed into a six-dimensional real value vector. In total, 65,536-dimensional raw 8-mer data and 70-dimension data, including sequence features, Fickett nucleotide features, GCcontent, EDP of 2-mer, physicochemical properties, and multi-scale structural features, were further processed using different feature learning and screening methods.

### 4.3. Feature Learning and Selection

In order to extract the most informative features and avoid overfitting, the feature learning based on the autoencoder and two different feature selection methods were applied to different types of features. Compared with other features, raw 8-mer features have a large number and more redundant data. Therefore, features were divided into two categories for feature learning and feature selection. Here, we simply refer to the two sets of features as fea.Tuple and fea.Bio, respectively, where the fea.Tuple contains the original 8-mer feature, and the fea.Bio contains the remaining features. For fea.Tuple, the filter filtering method based on binomial distribution was used, and for fea.Bio, after the autoencoder was used for advanced feature extraction, the recursive feature elimination algorithm was used. As shown in Figure 6, we denote the raw 8-mer features as fea.Tuple, and the rest of the features as fea.Bio. The strategy of the binomial distribution and iterative feature selection were used to select the most informative 8-mer features from fea.Tuple, and to obtain the filtered feature, i.e., the new fea.Tuple. For fea.Bio, firstly, two stacked encoders were used to learn higher level features of fea.Bio, and then the recursive feature elimination was used to further select the better features, and obtain the filtered feature, i.e., the new fea.Bio. After testing and evaluating different machine learning models, including support vector machines, random forests, logistic regression, XGBoost, and lightGBM, and deep learning methods, including DNN and CNN, the optimal model was selected. Finally, the new fea.Tuple and new fea.Bio were selected together to obtain the optimal machine learning scheme. The details of the feature learning and selection are shown as follows.

#### 4.3.1. Binomial Distribution Method and Iterative Feature Selection (IFS) Method for fea.Tuple

As the raw 8-mer feature contains 65,536 dimensions, there will be a lot of redundancy and noise, which will affect the performance of the model, leading to a dimensional disaster. Moreover, such a large-scale feature is not suitable for filtering with the recursive feature elimination algorithm, which may cause a memory exception on the machine. So, we used the feature selection method based on binomial distribution for feature selection [49,50].

The occurrence of a specific 8-mer in a certain lncRNA subcellular localization region is essentially random, and the prior probability of the certain 8-mer in each location was assumed to be:

(16) $$q_{j}=\frac{m_{j}}{M},$$

where $m_{j}$ refers to the number of one 8-mer fragment of the *j*-th location (j = 1, 2, 3, and 4, corresponding to four subcellular localization regions), and M represents to the total number of all 8-mer in the dataset.

Then, the probability of the *i*-th 8-mer in the *j*-th category $p_{\left(n_{ij}\right)}$ was calculated according to the prior probability:

(17) $$p_{\left(n_{ij}\right)}=\Sigma_{m=n_{ij}}^{N_{i}}\frac{N_{i}!}{m_{i\left(N_{i}-m\right)}}q_{j}^{m}{\left(1-q_{j}\right)}^{N_{i}-m},$$

where the number of occurrences of a given 8-mer on the *j*-th classification subset and benchmark dataset is represented by $n_{ij}$ and $N_{i}$, respectively. If the *i*-*i*th 8-mer on the *j*-th classification is not biologically significant, then the probability of it should be very small. So, we used *C* to represent their confidence level:

(18) $$C_{ij}=1-P\left(n_{ij}\right).$$

Since there are four classifications corresponding to four *C* values ($C_{i1},\;C_{i2},\;C_{i3},C_{i4}$), we took the maximum of them as the confidence value for each 8-mer:

(19) $$C_{i}=MAX\left(C_{i1},\;C_{i2},\;C_{i3},C_{i4}\right).$$

The optimal 8-mer subset was selected using iterative feature selection (IFS) according to the ranking of 8-mer confidence values from high to low. The 8-mer with the largest *C* value was used to test the model. Then, the 8-mer with the largest *C* value was added to the test model from the remaining 8-mer subset. The above steps were repeated until the model accuracy no longer increased. Finally, we obtaineed the feature set containing the optimal subset of 8-mer features, denoted as new fea.Tuple.

#### 4.3.2. Automatic Encoder and Recursive Feature Elimination (RFE) for fea.Bio

The fea.Bio was further processed through three steps.

Step 1: The extracted features were scaled by an automatic encoder with a symmetric network structure to obtain two tensor data of 32 and 64 dimensions.
Step 2: The recursive feature elimination algorithm was used to filter the 96-dimensional data encoded.
Step 3: In order to further eliminate the influence of the numerical scale and data noise between different features, and make it more suitable for model training, the normalization method was used to further process the data.

In order to enhance the representation ability of features, a symmetric autoencoder was used to learn higher-level features from 70-dimensional features. Autoencoder is a multi-layer neural network where the input and output layers represent the same meaning and have the same number of nodes. The autoencoder is composed of an encoder and a decoder. Here, we used an encoder with three layers of full connection layer, whose input was a 70-dimensional vector. The other two layers were 64- and 32-dimensional full connection layers, respectively. The corresponding encoder and decoder were composed of 32 and 64 dimensions and a 70-dimensional output.

By extracting the output of the middle layer, the 70-dimensional features were coded into a 32-dimensional vector. Then, the dimension of the self-encoder was adjusted to re-encode the 70-dimensional feature into a 64-dimensional vector, and finally a 96-dimensional feature vector was obtained.

In order to obtain the features with more power for identification, the recursive feature elimination algorithm [51,52,53] was used to further select the most informative features from the learned higher-level features. The stability of RFE depends largely on the underlying model used during iteration. Here, we used the XGBoost algorithm based on a parallel tree system [23]. As a boosting algorithm, XGBoost has a relatively strong ability to filter data. We used a system with a 0.1 learning rate and 50 subtrees to screen the features, and finally obtained a set of 32 dimensional features, denoted as new fea.Bio. The order that was eliminated in this process is the sort of features. By testing the 32-dimensional data with the 70-dimensional data extracted from the original data in the model, an effective improvement was obtained in the final results.

#### 4.3.3. Model Selection

To further explore these features, we tested a variety of machine learning models, including traditional classification models, such as support vector machines, random forests, logistic regression, XGBoost, and lightGBM, and deep learning methods, including DNN and CNN.

Through comprehensive evaluation of the performance of different feature combinations and machine learning models with 10-fold cross validation, SVM was used to construct lncLocation.

### 4.4. Performance Evaluation

For model comparison, we adopted 10-fold cross validation. The initial sampling was divided into 10 subsamples, one single subsample was retained as the data for verification of the model, and the other 9 samples were used for training. The cross-validation was repeated 10 times, one for each subsample, and the results averaged 10 times, resulting in a single estimate.

To evaluate the efficiency of one model for subcellular localization of lncRNAs, we introduced some criteria, including accuracy, precision, recall, and F1-score, which can be formulated as follows:

{(20)Accuracy=Num(pred=lable)Num(pred),(21)Precision(i)=TP(i)TP(i)+FP(i),(22)Recall(i)=TP(i)TP(i)+FN(i),(23)F1=1n∑i=1n2×Precision(i)×Recall(i)Precision(i)+Recall(i),

where $TP^{\left(i\right)}$, $\;FP^{\left(i\right)}$, and $FN^{\left(i\right)}$ represent the true positive, false positive, and false negative of the ith class, respectively.

## Figures and Tables

**Table 1:** The comparison of basic features on different models.

Feature	Method	Precision	Recall	F-Score	Accuracy
K-tuple features	**Autoencoder(8-mer) + SVM**	0.3622	0.2709	0.2388	**0.6650**
	Autoencoder(8-mer) + RF	0.3558	0.2701	0.2379	0.6654
	Autoencoder(8-mer) + LR	0.2081	0.2506	0.2040	0.6460
	Autoencoder(8-mer) + XGBoost	0.3271	0.2741	0.2487	0.6559
	Autoencoder(8-mer) + LightGBM	0.3031	0.2649	0.2308	0.6573
	Autoencoder(8-mer) + EDP + SVM	0.3888	0.2682	0.2331	0.6647
	**Autoencoder(8-mer) + EDP + RF**	0.2938	0.2712	0.2376	**0.6661**
	Autoencoder(8-mer) + EDP + LR	0.3787	0.2906	0.2790	0.6430
	Autoencoder(8-mer) + EDP + XGBoost	0.3315	0.2716	0.2464	0.6522
	Autoencoder(8-mer) + EDP + LightGBM	0.2946	0.2668	0.2325	0.6606
Properties of open reading frame	SVM	0.1622	0.2500	0.1967	0.6488
	RF	0.3596	0.2863	0.2748	0.6387
	**LR**	0.2641	0.2575	0.2120	**0.6598**
	XGBoost	0.3023	0.2644	0.2404	0.6265
	LightGBM	0.2477	0.2526	0.2098	0.6457
Fickett nucleotide features	SVM	0.2843	0.2560	0.2120	0.6497
	**RF**	0.3108	0.2814	0.2633	**0.6570**
	LR	0.1985	0.2633	0.2167	0.6539
	XGBoost	0.3874	0.2946	0.2910	0.6366
	LightGBM	0.3636	0.2904	0.2844	0.6338
Physicochemical properties	SVM	0.3232	0.2564	0.2098	0.6549
	RF	0.2740	0.2673	0.2495	0.6127
	LR	0.3449	0.2629	0.2229	0.6636
	XGBoost	0.2752	0.2649	0.2399	0.6268
	**LightGBM**	0.4111	0.3913	0.3728	**0.7018**
Mutli-scale secondary structures	**SVM**	0.5076	0.4590	0.4356	**0.7169**
	RF	0.4204	0.4171	0.4000	0.6927
	LR	0.2648	0.2574	0.2133	0.6576
	XGBoost	0.4318	0.4122	0.4023	0.6928
	LightGBM	0.4248	0.4040	0.3870	0.7042

For testing purposes, the autoencoder converts 65,536-dimensional 8-mer data into 128-dimensional output. The encoding layer consists of an input with 65,536 dimensions and three intermediate layers with nodes of 4096, 1024, and 256, respectively. The decoding layer corresponds to the encoding layer, and finally converts the 8-mer sequence into the 128-dimensional real value vector. EDP represents the combination of the EDP of the 2-mer and the EDP of the ORF.

**Table 2:** The comparison between lncLocation and state-of-the-art predictor.

Location	lncLocation			iLoc-lncRNA			lncLocator		
	Precision	Recall	Overall	Precision	Recall	Overall	Precision	Recall	Overall
			Accuracy			Accuracy			Accuracy
Nucleus	0.9583	0.7419	0.8778	0.9759	0.7756	0.8672	0.9217	0.3815	0.6650
Cytoplasm	0.8500	1.0000		0.6768	0.9906		0.3636	0.8801	
Ribosome	1.0000	0.5556		0.9983	0.4651		0.9753	0.0700	
Exosome	1.0000	0.3333		1.0000	0.1667		0.9727	0.0400	

**Table 3:** Benchmark lncRNA subcellular localization dataset.

Subcellular Localizations	Support Number
Cytoplasm	426
Nucleus	156
Ribosome	43
Exosome	30

## Supplementary Materials

The following are available online at https://www.mdpi.com/1422-0067/21/19/7271/s1. Supplementary Table S1. New Fea.Bio training results on each model. Supplementary Table S2. Connecting new Fea.Tuple and new Fea.Bio training results on each model. Supplementary Table S3. New Fea.Tuple training results on each model. Supplementary Data S1. The benchmark dataset of lncLocation. Supplementary Data S2. Subcellular localization prediction results of human lncRNA genome based on lncLocation. Supplementary Data S3. Data sets used for human lncRNA genome prediction based on lncLcation.

## Availability and Implementation

A convenient web is available at http://lnclocation.nat100.top/ and software packages suitable for Windows and Linux systems are available at https://github.com/FengSY-JLU/Core-lncLocation/.
