# Supplementary material for: LncLocation: Efficient Subcellular Location Prediction of Long Non-Coding RNA-Based Multi-Source Heterogeneous Feature Fusion
Source: Int J Mol Sci. 2020 Oct 1;21(19):7271. doi: 10.3390/ijms21197271 (PMC7582431; doi:10.3390/ijms21197271)
Supplement: Supplementary file 1 [file ijms-21-07271-s001.zip › ijms-924790-supplementary/Supplementary Table S1.docx]

**Supplementary Table S1. New Fea.Bio training results on each model.**

| Model | Precision | Recall | F1-Score | Accuracy |
| --- | --- | --- | --- | --- |
| SVM | 0.58 | 0.46 | 0.49 | 0.78 |
| RF | 0.55 | 0.46 | 0.48 | 0.78 |
| LR | 0.30 | 0.27 | 0.23 | 0.66 |
| DNN | 0.37 | 0.36 | 0.35 | 0.63 |
| CNN | 0.33 | 0.43 | 0.31 | 0.61 |
| XGboost | 0.57 | 0.47 | 0.50 | 0.78 |
| LightGBM | 0.50 | 0.42 | 0.43 | 0.75 |
