# Supplementary material for: LncLocation: Efficient Subcellular Location Prediction of Long Non-Coding RNA-Based Multi-Source Heterogeneous Feature Fusion
Source: Int J Mol Sci. 2020 Oct 1;21(19):7271. doi: 10.3390/ijms21197271 (PMC7582431; doi:10.3390/ijms21197271)
Supplement: Supplementary file 1 [file ijms-21-07271-s001.zip › ijms-924790-supplementary/Supplementary Table S2.docx]

**Supplementary Table S2. Connecting new Fea.Tuple and new Fea.Bio training results on each model.**

| Model | Precision | Recall | F1-Score | Accuracy |
| --- | --- | --- | --- | --- |
| SVM | 0.82 | 0.62 | 0.67 | 0.87 |
| RF | 0.48 | 0.32 | 0.32 | 0.70 |
| LR | 0.29 | 0.28 | 0.25 | 0.65 |
| DNN | 0.32 | 0.34 | 0.32 | 0.56 |
| CNN | 0.42 | 0.45 | 0.39 | 0.60 |
| XGboost | 0.60 | 0.39 | 0.41 | 0.72 |
| LightGBM | 0.47 | 0.36 | 0.37 | 0.71 |
