# Supplementary material for: LncLocation: Efficient Subcellular Location Prediction of Long Non-Coding RNA-Based Multi-Source Heterogeneous Feature Fusion
Source: Int J Mol Sci. 2020 Oct 1;21(19):7271. doi: 10.3390/ijms21197271 (PMC7582431; doi:10.3390/ijms21197271)
Supplement: Supplementary file 1 [file ijms-21-07271-s001.zip › ijms-924790-supplementary/Supplementary Table S3.docx]

**Supplementary Table S3. New Fea.Tuple training results on each model.**

| Model | Precision | Recall | F1-Score | Accuracy |
| --- | --- | --- | --- | --- |
| SVM | 0.70 | 0.55 | 0.58 | 0.85 |
| RF | 0.52 | 0.34 | 0.34 | 0.70 |
| LR | 0.16 | 0.25 | 0.19 | 0.65 |
| DNN | 0.42 | 0.26 | 0.22 | 0.67 |
| CNN | 0.22 | 0.27 | 0.24 | 0.64 |
| XGboost | 0.52 | 0.32 | 0.32 | 0.69 |
| LightGBM | 0.45 | 0.31 | 0.31 | 0.68 |
